# Genetic resonance: dissecting the heritability and genetic correlations of human hearing acuity

**DOI:** 10.1093/g3journal/jkae292

**Published:** 2024-12-12

**Authors:** Jerry A Duran, W Scott Watkins, Deborah W Neklason, Lynn B Jorde

**Affiliations:** Department of Human Genetics, The University of Utah, Salt Lake City, UT 84112, USA; Department of Human Genetics, The University of Utah, Salt Lake City, UT 84112, USA; Department of Internal Medicine, The University of Utah Medical School, The University of Utah, Salt Lake City, UT 84112, USA; Department of Human Genetics, The University of Utah, Salt Lake City, UT 84112, USA

**Keywords:** heritability, family studies, genetic correlation, hearing acuity

## Abstract

This study presents a frequency-specific, pedigree-based assessment of hearing acuity heritability. We analysed 34 Utah Centre d'Etude du Polymorphisme Humain (CEPH) pedigrees comprising 464 individuals, using whole-genome sequencing (WGS) and audiometric evaluations. Our analysis reveals a general decline in narrow-sense heritability as frequency increases. When calculated using the Sequential Oligogenic Linkage Analysis Routines (SOLAR) software package, narrow-sense heritability estimates drop from 51% at 250 Hz to 30% at 8000 Hz. Genetic correlations (Rho(G)), the degree to which genetic variation in one trait correlates with another, are higher for hearing acuity at similar frequencies. For example, Rho(G) between 250 and 500 Hz is 0.80, and Rho(G) between 6000 and 8000 Hz is 0.91. In contrast, frequencies distant from each other exhibit decreased Rho(G) with a genetic correlation of only 0.21 for hearing acuity at frequencies 250 and 8000 Hz. This assessment of the genetics underlying hearing acuity in a family-based design provides new details of genetic factors influencing hearing acuity in a frequency-specific approach.

## Introduction

The genetics of hearing impairment (HI) has gained significant attention in recent years, with the World Health Organization estimating that the number of individuals affected by HI will increase to 2.5 billion by 2050 (World Health Organization 2021). The causes of HI and deafness are multifaceted, encompassing both genetic and environmental factors, with genetics accounting for 50–60% of the variation (McDermott *et al*. 2019; Mathias *et al*. 2022). By studying hearing across the full range of hearing ability, rather than HI alone, we can better understand the genetic mechanisms that maintain healthy auditory function (Taylor 2003; Afghah *et al*. 2023).

Hearing acuity is measured as the minimum volume necessary to perceive sound and is typically measured from 500 to 16,000 Hz. Previous research on the genetics of hearing acuity has been primarily centred on age-related hearing decline (Viljanen *et al*. 2009; Duan *et al*. 2019), known as presbycusis. Twin studies have estimated the heritability of hearing acuity across low, middle, and high-frequency ranges (Viljanen *et al*. 2009; Duan *et al*. 2019). Duan *et al*. estimated the heritability of age-related hearing loss, ranging from 1.65% at 500 Hz to 54% at 12,500 Hz. Viljanen *et al*. (2009) estimated heritability, averaged across low, middle, and high frequencies, at greater than 60%. Genetic factors might vary distinctly across frequencies (Shah *et al*. 2005; Pavlenkova *et al*. 2021), with DNA variants affecting either low- or high-frequency HI (Psillas *et al*. 2019; Ma *et al*. 2022; Oh *et al*. 2023).

Heritability estimates are typically derived from twin and family studies but can be biased by shared environmental influences and epistatic interactions (Elks *et al*. 2012; Smith *et al*. 2023). Narrow-sense heritability (*h*²) measures the proportion of phenotypic variance explained by additive genetic factors and can be estimated from correlations among family members (Athanasiadis *et al*. 2020).

Whole-genome sequencing (WGS) comprehensively captures most of the genetic variation within a genome. In contrast to whole exome sequencing or single nucleotide polymorphism (SNP) arrays, WGS can identify genetic variants influencing heritability in most regions of the genome (Ohnmacht *et al*. 2020; Sullivan *et al*. 2023). Coupled with family data, WGS has the potential to yield valuable new insights into the genetics of hearing acuity (Manichaikul *et al*. 2010; Guan *et al*. 2022).

Here, we analyse genetic and hearing acuity data from 34 CEPH pedigrees. These families played a central role in creating a genetic linkage map as part of the Human Genome Project (Dausset *et al*. 1990). They later contributed to the Haplotype Map Project (The International HapMap Consortium 2003) and the 1000 Genomes Project (Auton *et al*. 2015). Six hundred and three members of these three-generation families underwent WGS, providing a basis to study various types of de novo mutations and their health consequences (Feusier *et al*. 2019; Sasani *et al*. 2019; Cawthon *et al*. 2020; Belyeu *et al*. 2021; Steely *et al*. 2022). Extensive phenotype data have also been collected from family members, including hearing acuity measurements at frequencies ranging from 250 to 8000 Hz (Prescott *et al*. 2008).

Family-based designs are helpful in investigating the role of genetic variation in phenotypic variability. The genetic homogeneity within families increases the potential to detect genetic factors that may influence hearing acuity (Abdellaoui *et al*. 2023). Our study investigates genetic variation associated with frequency-specific hearing acuity using WGS combined with audiometric data and finds that, in general, as frequency increases, heritability estimates decrease. In addition, genetic correlations are more similar for neighbouring frequencies than for frequencies that are more distant from one another. This assessment of the genetics impacting hearing acuity in a family-based design provides new details of factors influencing hearing in a frequency-specific approach and provides the framework for future research to further identify the genetics modulating hearing.

## Methods

### Cohort

Research participants provided informed consent for research conducted as part of the Utah Genetic Reference Project, which was approved by the University of Utah's Institutional Review Board IRB# 0080145 (Prescott *et al*. 2008). The cohort analysed here comprises 464 individuals from 34 Utah CEPH families, ranging from 18 to 91 years old. The families consist of three generations and have an average of eight siblings in the third generation, ranging from 6 to 12 siblings per family. There are 24 individuals in generation 1, 100 in generation 2, and 340 in generation 3.

### Whole-genome sequencing

CEPH family members underwent WGS at 30× coverage, and data were processed to variant call files (VCFs), as described by Sasani *et al*. (2019) National Center for Biotechnology Information, 2019. Genetic variants in the VCFs were processed to remove insertion–deletion variants and multi-allelic SNPs. The remaining bi-allelic SNPs were filtered to exclude those with minor allele frequencies (MAF) less than 0.10 and those not in Hardy–Weinberg equilibrium, using a *P*-value threshold of less than 1 × 10^−6^. The SNPs were then pruned for interdependence by omitting one member of each pair of SNPs with linkage disequilibrium values (*r*^2^) greater than 0.2 using PLINK v1.9 (Purcell *et al*. 2007). SNPs with Mendelian errors were omitted. After quality control and filtering, 428,537 SNPs were available for analysis.

### Audiometric data collection and preparation

Research participants underwent hearing tests at the University of Utah audiology clinic using air conduction audiometry with Grason Stadler GSI 61 audiometers (Saunders *et al*. 2000). The audiometer evaluated hearing acuity at eight different frequencies: 250, 500, 1000, 2000, 3000, 4000, 6000, and 8000 Hz. In addition, pure-tone and high-frequency averages were calculated using the averages of 250, 500, 1000, 2000, and 3000 Hz, 4000, 6000, and 8000 Hz, respectively. The best hearing ear threshold was used for analysis. Because our study assessed normal hearing, individuals were excluded from further analysis if data from both ears were unavailable at any frequency. Our cohort consists of 230 females and 234 males. At the time of the hearing assessment, the average age of the cohort was 40 years, the maximum age was 91 years, and the minimum age was 18 years. Audiometric values were normalized using the Sequential Oligogenic Linkage Analysis Routines (SOLAR) v8.3.1 software package, using inverse rank-based normalization for individual frequencies before downstream analysis (Almasy and Blangero 1998).

### Heritability analysis

The significance of age and sex in explaining observed phenotypic variation within families was assessed using SOLAR, which carries out covariate screening using a mixed-effects model. Age and sex were included as covariates for subsequent analysis. In addition, SOLAR addresses the polygenicity of traits and is adept at handling complex family structures, especially when assessing correlations among family members.

SOLAR uses identity-by-descent calculations to derive a genomic relationship matrix (GRM) from either individual SNP genotype data or user-supplied pedigree relationships. The SOLAR software package was used to estimate narrow-sense heritability for each frequency and for pure-tone and high-frequency averages. The Pearson correlation coefficient for heritability estimates derived from SNP-based and pedigree-based GRMs was 0.99.

### Genetic correlation analysis

SOLAR uses a variance components approach to estimate the genetic correlation, Rho(G), between a pair of traits (in this case, hearing acuity at different frequencies). Rho(G) denotes the degree to which SNP variation underlying one trait correlates with SNP variation in another and is thus a measure of pleiotropy (Almasy and Blangero 2010). The variance components model indirectly estimates Rho(E), an inferred correlation between traits due to nongenetic (shared environmental) factors. Because Rho(E) represents a residual effect after taking the effect of genetic variation on a phenotype into account, it also includes error variation (Almasy and Blangero 1998; Mathias *et al*. 2022). We estimated Rho(G) and Rho(E) for all pairwise combinations of hearing acuity thresholds at each of eight different frequencies. These estimates were generated for (i) all relationships (generations 1, 2, and 3) (ii) parent–offspring relationships (generations 2 and 3), and (iii) sibling relationships (generation 3). Confidence intervals were compared to identify significant differences in Rho(G) and Rho(E) between the parent–offspring and sibling groups.

## Results

### Narrow-sense heritability

As individuals age, hearing acuity tends to decline, and this effect is more pronounced in males, occurring earlier and to a greater extent (Nolan 2020; Aloufi *et al*. 2023). Accordingly, we evaluated the significance of age, sex, and the combined effect of age and sex on hearing acuity at individual frequencies, and pure-tone and high-frequency averages. Age was identified as a significant covariate (*P* < 0.05) at every frequency and for the pure-tone and high-frequency averages (Table 1). In our cohort, males displayed decreased hearing acuity, and sex was identified as a significant covariate at frequencies of 2000, 3000, 4000, and 6000 Hz, as well as in both averaged measures (*P* < 0.05). The combined effect of age and sex was just above the significance threshold at all frequencies and pure-tone and high-frequency averages. Subsequent analysis adjusted for these identified covariates.

**Table 1.** Comparison of sporadic and polygenic models across auditory frequencies.

Frequency (Hz)	Log-likelihood of a sporadic model	Log-likelihood of a polygenic model	Chi-squared test value, df = 1	*P*-value	Covariates
250	−193.76	−142.66	102.91	2.51 × 10^−24^	Age
500	−179.70	−143.79	71.81	1.18 × 10^−17^	Age
1000	−153.35	−131.98	42.75	3.10 × 10^−11^	Age
2000	−147.36	−105.72	83.33	3.48 × 10^−20^	Age, sex
3000	−122.15	−107.18	29.96	2.21 × 10^−08^	Age, sex
4000	−102.29	−91.41	21.57	1.60 × 10^−06^	Age, sex
6000	−78.94	−63.08	31.72	8.89 × 10^−9^	Age, sex
8000	−92.83	−76.97	31.72	8.86 × 10^−09^	Age
Pure-tone average	−137.58	−96.08	83.01	4.09 × 10^−20^	Age, sex
High frequency average	−64.08	−43.18	41.81	7.1 × 10^−11^	Age, sex

Log-likelihood values for both sporadic and polygenic models of hearing acuity, evaluated across different auditory frequencies. Chi-squared test values for the polygenic model (the best-fitting model) are shown, df = 1, and their corresponding *P*-values are provided as indicators of the goodness of fit for each model. The log-likelihood of a sporadic model indicates the model's fit under the assumption that the trait is not influenced by genetic inheritance. The log-likelihood of a polygenic model evaluates the fit of a model that assumes the trait in question is influenced by multiple genetic loci. The Chi-squared test validates which model is a better fit for the data, and the *P*-value indicates the significance of the Chi-squared test. Additionally, covariates employed in the models are indicated in the covariate column.

**Fig. 1.**
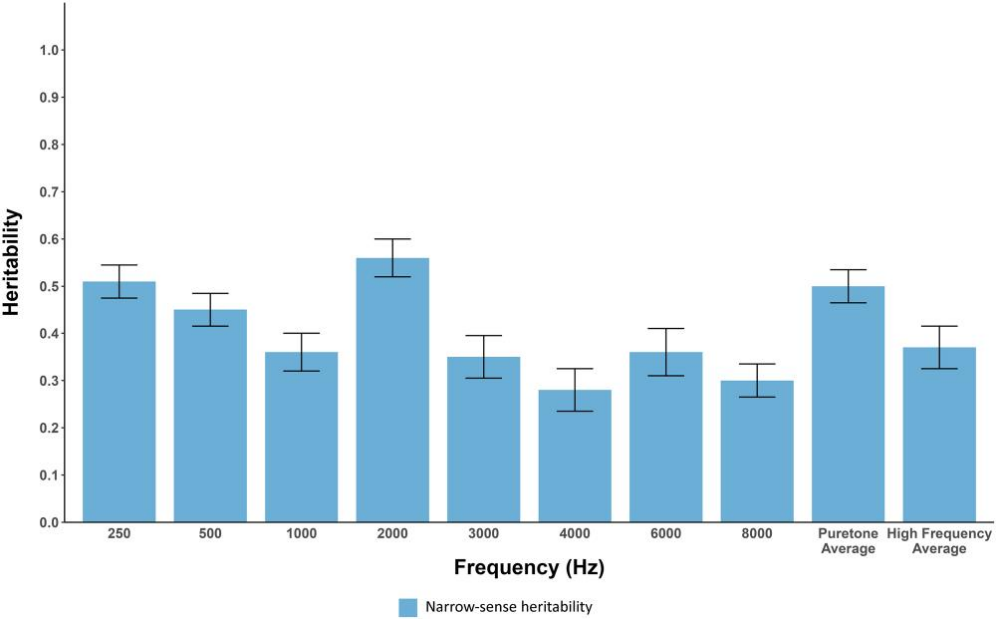
Hearing acuity heritability estimates. Heritability estimates were derived for hearing acuity at different frequencies and two frequency averages (*X*-axis) with standard errors indicated. The pure-tone average is the average of hearing thresholds at 250, 500, 1000, and 2000 Hz. The high-frequency average is the average of hearing thresholds at 3000, 4000, 6000, and 8000 Hz.

The polygenic nature of hearing acuity at each frequency was assessed using SOLAR (Almasy and Blangero 1998) (see Table 1). A significant polygenic contribution to hearing acuity was detected at all individual frequencies and for the pure-tone and high-frequency averages, consistent with prior studies (Hoffmann *et al*. 2016; Ingham *et al*. 2020). Narrow-sense heritability was estimated using SOLAR's maximum likelihood algorithm. Narrow-sense heritability ranged from 51% (95% confidence interval = 37.3–64.7%) at 250 Hz to 30% (95% confidence interval = 16.3–45.6%) at 8000 Hz, with the highest heritability estimate, 56%, at 2000 Hz (95% confidence interval = 40.3–71.7%). Heritability estimates generally decreased with increasing frequency, although the trend was not statistically significant (Fig. 1, Supplementary Table 1 and Fig. 1).

### Genetic correlations are higher at neighbouring frequencies

**Fig. 2.**
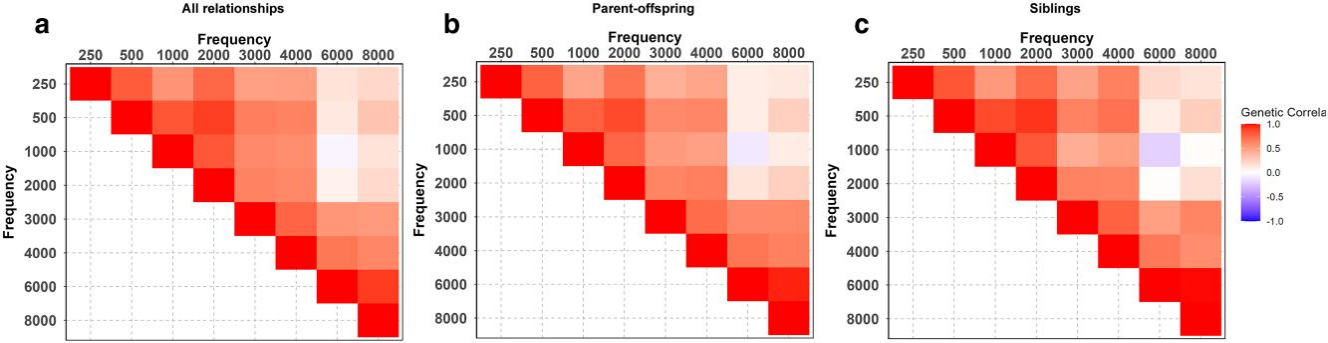
Assessment of genetic correlation across familial relationships. Genetic correlation, Rho(G), is evaluated for: a) all relationships; generations 1, 2, and 3; b) parent and offspring relationships, generations 2 and 3; and c) sibling relationships in generation 3. The diagonal shows self-correlation. The intensity of the colour reflects the degree of correlation between the two frequencies—darker red shades denote a stronger positive correlation, white indicates no correlation, and blue indicates a negative correlation.

The genetic correlation (Rho(G)) analysis revealed stronger correlations among frequencies closer together within the range of measured frequencies. For example, the adjacent frequencies 250 and 500 Hz had a Rho(G) of 0.80. A similarly high Rho(G) (0.91) was observed between 6000 and 8000 Hz (Fig. 2a and Supplementary Table 2). In contrast, frequencies more distantly separated, such as 250 and 8000 Hz, had a much smaller Rho(G) = (0.21). This pattern was consistent across most frequency comparisons, providing evidence that a larger difference in frequency yields a lower genetic correlation.

Prior studies have indicated that shared environmental factors can influence both genetic correlation and heritability estimates (Visscher *et al*. 2008; Van Dongen *et al*. 2012; van Rheenen *et al*. 2019). It has been suggested that higher genetic correlations among siblings, compared to parents and offspring, could signify the influence of shared environmental effects (Baselmans *et al*. 2021). To test this hypothesis, we compared genetic correlations between siblings and parent and offspring relationships (Figs. 2b and 2c). Comparing 95% confidence intervals, we found no significant differences among parent–offspring and sibling correlations (Supplementary Fig. 3). A negative Rho(G) was identified in the parent–offspring and sibling pairs between frequencies 1000 and 6000 Hz. Negative Rho(G) values could indicate that the genetic variation impacting hearing acuity at one frequency may inversely impact hearing acuity at another frequency.

We further assessed the impact of shared environmental factors using the environmental correlation, Rho(E). It is expected that that siblings will have a higher Rho(E) than parents and offspring because they tend to share more of their environment, especially if they are close in age and grow up in the same household. We compared Rho(E) values between parent–offspring and sibling pairs (Supplementary Fig. 2 and Table 3) and found that there were no significant differences between parent–offspring and sibling groups (Supplementary Fig. 4). However, there was, on average, an unexpected but non-significant increase in Rho(E) in the parent–offspring compared to sibling groups.

## Discussion

Our study reports the first frequency-specific hearing acuity heritability estimates in families. Our findings demonstrate an inverse trend between heritability estimates and frequency, contrasting with those reported in a study of geriatric Finnish female twin pairs (Viljanen *et al*. 2009), where heritability estimates exceeded 60% across averaged low, medium, and high frequencies. This finding may reflect the well-known tendency for twin studies to overestimate heritability due to violation of assumptions, such as equally shared environments in monozygotic and dizygotic twins (Van Dongen *et al*. 2012). Our results also differ from a Chinese twin study focusing on age-related HI in middle-aged and elderly participants (Duan *et al*. 2019), which reported increased heritability for frequencies above 2000 Hz and decreased heritability at 500 and 1000 Hz. This could reflect differences in the ages of the cohorts as well as differences in environmental exposures or lifestyle factors. Additionally, genetic factors influencing hearing loss may vary across populations due to differences in genetic backgrounds.

Our analysis showed that as frequency increases, heritability decreases. This is thought to be due to environmental effects on hearing, such as prolonged exposure to loud noises, which disproportionately impact high-frequency hearing acuity (Gopinath et al. 2021). Destefano *et al*. (2003) employed a pedigree-based approach utilizing SOLAR to assess the heritability of age-related HI for middle and low frequencies using the Framingham Heart Study data. They reported heritability averages of 36 and 45%, respectively. Our study, which focused on frequency-specific hearing acuity, produced heritability estimates of 37 and 51% when averaging the same frequency ranges.

The genetic correlation analysis revealed that genetic variation that impacts hearing acuity at one frequency is likely to impact hearing acuity at neighbouring frequencies. The closer the frequencies were together, the higher their genetic correlation, suggesting common biology for neighbouring frequencies. The pattern is consistent across most of the Rho(G) analyses. Biologically, the cochlea is tonotopically organized, meaning that specific frequencies resonate along certain parts of the cochlea, with higher frequencies at the base and lower frequencies at the apex (Hudspeth 1985). This helps to explain the frequency-specific patterns observed in our study (Shearer *et al*. 2023). Other research provides evidence that specific genetic variation may affect specific frequency ranges. For example, mutations in the gene that encodes cadherin 23 (*CDH23*) show an increased frequency in persons with high-frequency hearing loss (Mizutari *et al*. 2015).

We observe a consistent genetic influence across various frequencies, and heritability estimates are lower at higher frequencies. Our comparison of genetic correlations within parent–offspring and sibling pairs did not suggest statistically significant effects of shared environmental factors on hearing acuity estimates, although statistical power is limited by our sample size. In summary, our findings highlight the complexity of genetic contributions to hearing acuity and underscore the importance of considering frequency-specific analyses.

## Supplementary Material

jkae292_Supplementary_Data

## Data Availability

The data discussed are under protected access at the following locations. Phenotype and family data can be requested through the Utah Genome Project https://uofuhealth.utah.edu/center-genomic-medicine/research/ceph-resources. Genomic data are available through the dbGaP database. Genome Sequencing of Large, Multigenerational CEPH/Utah Families Study Accession: phs001872.v1.p1 Command line and R code with explanations can be found at the following GitHub location: https://github.com/FishingNerd/hearing_acuity Supplemental material available at G3 online.
