# Supplementary material for: Genetic resonance: dissecting the heritability and genetic correlations of human hearing acuity
Source: G3 (Bethesda). 2024 Dec 12;15(2):jkae292. doi: 10.1093/g3journal/jkae292 (PMC11797064; doi:10.1093/g3journal/jkae292)
Supplement: jkae292_Supplementary_Data [file jkae292_supplementary_data.zip › Supplemental_Material_and_Figure_Legends_G3-2024-405327.docx]

**Supplemental figure legends**

**Supplemental Figure 1. 95% confidence intervals for narrow-sense-heritability.**

**Supplemental Figure 2. Environmental variance correlation estimates.**

Rho(E) values are shown for A) all relationships: generations 1, 2, and 3, B) parent and offspring relationships: generations 2 and 3, and C) sibling relationships in generation 3. The diagonal signifies an environmental coefficient of one. The intensity of the red color reflects the degree of environmental influence on each pair of frequencies. Darker red shades denote a stronger positive correlation, and white indicates little to no environmental effect on generic correlation estimates.

**Supplemental Figure 3. Genetic correlation (Rho(G)) 95% confidence intervals**

Individual plots display the genetic correlation at various frequency comparisons for: all relationships: generations 1, 2, and 3; parent and offspring relationships: generations 2 and 3; and sibling relationships: generation 3.

**Supplemental Figure 4. Environmental correlation (Rho(E)) 95% confidence intervals**

Individual plots display (Rho(E)) at various frequency comparisons for all relationships: generations 1, 2, and 3; parent and offspring relationships: generations 2 and 3; and sibling relationships: generation 3.

**Supplemental Tables**

| Frequency | Narrow-sense heritability | Standard error |
| --- | --- | --- |
| 250 | 0.51 | 0.07 |
| 500 | 0.45 | 0.07 |
| 1000 | 0.36 | 0.08 |
| 2000 | 0.56 | 0.08 |
| 3000 | 0.35 | 0.08 |
| 4000 | 0.28 | 0.07 |
| 6000 | 0.36 | 0.10 |
| 8000 | 0.30 | 0.08 |
| Pure tone Average | 0.50 | 0.1 |
| High Frequency Average | 0.36 | 0.07 |

**Supplemental Table 1. Comparison of heritability estimates for hearing acuity at different frequencies.**

| Frequency comparison | All Generations  Rho(G) | S.E. | Parent-offspring  Rho(G) | S.E. | Sibling  Rho(G) | S.E. |
| --- | --- | --- | --- | --- | --- | --- |
| 250\|500 | 0.80 | 0.07 | 0.77 | 0.08 | 0.82 | 0.08 |
| 250\|1000 | 0.55 | 0.12 | 0.47 | 0.14 | 0.52 | 0.16 |
| 250\|2000 | 0.75 | 0.09 | 0.70 | 0.10 | 0.74 | 0.11 |
| 250\|3000 | 0.49 | 0.14 | 0.41 | 0.16 | 0.47 | 0.18 |
| 250\|4000 | 0.50 | 0.15 | 0.47 | 0.17 | 0.64 | 0.16 |
| 250\|6000 | 0.15 | 0.16 | 0.10 | 0.17 | 0.20 | 0.21 |
| 250\|8000 | 0.21 | 0.16 | 0.12 | 0.17 | 0.15 | 0.22 |
| 500\|1000 | 0.82 | 0.08 | 0.78 | 0.10 | 0.86 | 0.08 |
| 500\|2000 | 0.90 | 0.59 | 0.86 | 0.08 | 0.93 | 0.06 |
| 500\|3000 | 0.65 | 0.12 | 0.60 | 0.14 | 0.63 | 0.16 |
| 500\|4000 | 0.63 | 0.14 | 0.61 | 0.16 | 0.71 | 0.15 |
| 500\|6000 | 0.12 | 0.17 | 0.10 | 0.19 | 0.09 | 0.23 |
| 500\|8000 | 0.30 | 0.16 | 0.25 | 0.18 | 0.26 | 0.22 |
| 1000\|2000 | 0.82 | 0.07 | 0.76 | 0.10 | 0.82 | 0.08 |
| 1000\|3000 | 0.60 | 0.14 | 0.52 | 0.16 | 0.42 | 0.19 |
| 1000\|4000 | 0.58 | 0.16 | 0.49 | 0.19 | 0.49 | 0.20 |
| 1000\|6000 | -0.05 | 0.19 | -0.10 | 0.20 | -0.19 | 0.23 |
| 1000\|8000 | 0.15 | 0.19 | 0.10 | 0.20 | 0.02 | 0.24 |
| 2000\|3000 | 0.63 | 0.12 | 0.62 | 0.12 | 0.63 | 0.15 |
| 2000\|4000 | 0.61 | 0.14 | 0.65 | 0.14 | 0.62 | 0.16 |
| 2000\|6000 | 0.07 | 0.18 | 0.14 | 0.18 | 0.01 | 0.23 |
| 2000\|8000 | 0.20 | 0.17 | 0.25 | 0.18 | 0.17 | 0.23 |
| 3000\|4000 | 0.76 | 0.11 | 0.73 | 0.12 | 0.77 | 0.12 |
| 3000\|6000 | 0.54 | 0.15 | 0.59 | 0.15 | 0.49 | 0.19 |
| 3000\|8000 | 0.52 | 0.16 | 0.60 | 0.15 | 0.62 | 0.17 |
| 4000\|6000 | 0.68 | 0.12 | 0.69 | 0.13 | 0.68 | 0.14 |
| 4000\|8000 | 0.61 | 0.14 | 0.64 | 0.15 | 0.58 | 0.18 |
| 6000\|8000 | 0.91 | 0.08 | 0.97 | 0.07 | 0.99 | 0.06 |

**Supplemental Table 2. Genetic correlation and standard errors.** Genetic correlations are shown for A) all relationships: generations 1, 2, and 3, B) parent and offspring relationships: generations 2 and 3, and C) sibling relationships in generation 3.

| Frequency comparison | All generations | | Parent-offspring | | Siblings | |  |
| --- | --- | --- | --- | --- | --- | --- | --- |
|  |  |  |  |  |  |  |  |
|  | Rho(E) | S.E. | Rho(E) | S.E. | Rho(E) | S.E. |  |
| 250\|500 | 0.63 | 0.05 | 0.53 | 0.06 | 0.25 | 0.24 |  |
| 250\|1000 | 0.30 | 0.23 | 0.30 | 0.19 | 0.29 | 0.21 |  |
| 250\|2000 | 0.03 | 0.09 | 0.07 | 0.10 | -0.24 | 0.19 |  |
| 250\|3000 | 0.15 | 0.08 | 0.19 | 0.08 | 0.13 | 0.20 |  |
| 250\|4000 | 0.13 | 0.08 | 0.18 | 0.08 | 0.02 | 0.20 |  |
| 250\|6000 | 0.23 | 0.08 | 0.30 | 0.09 | 0.36 | 0.19 |  |
| 250\|8000 | 0.22 | 0.08 | 0.27 | 0.09 | 0.37 | 0.19 |  |
| 500\|1000 | 0.49 | 0.06 | 0.49 | 0.07 | 0.28 | 0.19 |  |
| 500\|2000 | 0.25 | 0.08 | 0.23 | 0.09 | -0.07 | 0.32 |  |
| 500\|3000 | 0.18 | 0.08 | 0.20 | 0.08 | 0.11 | 0.18 |  |
| 500\|4000 | 0.09 | 0.08 | 0.12 | 0.08 | -0.07 | 0.19 |  |
| 500\|6000 | 0.17 | 0.09 | 0.18 | 0.10 | 0.17 | 0.18 |  |
| 500\|8000 | 0.20 | 0.08 | 0.21 | 0.09 | 0.19 | 0.17 |  |
| 1000\|2000 | 0.50 | 0.06 | 0.52 | 0.07 | 0.38 | 0.18 |  |
| 1000\|3000 | 0.26 | 0.07 | 0.30 | 0.08 | 0.34 | 0.15 |  |
| 1000\|4000 | 0.16 | 0.07 | 0.23 | 0.08 | 0.20 | 0.15 |  |
| 1000\|6000 | 0.26 | 0.08 | 0.31 | 0.09 | 0.46 | 0.19 |  |
| 1000\|8000 | 0.33 | 0.07 | 0.37 | 0.08 | 0.53 | 0.16 |  |
| 2000\|3000 | 0.46 | 0.07 | 0.48 | 0.07 | 0.40 | 0.15 |  |
| 2000\|4000 | 0.28 | 0.08 | 0.29 | 0.09 | 0.15 | 0.18 |  |
| 2000\|6000 | 0.26 | 0.09 | 0.24 | 0.10 | 0.30 | 0.20 |  |
| 2000\|8000 | 0.30 | 0.08 | 0.29 | 0.09 | 0.32 | 0.18 |  |
| 3000\|4000 | 0.50 | 0.05 | 0.53 | 0.06 | 0.43 | 0.11 |  |
| 3000\|8000 | 0.38 | 0.07 | 0.37 | 0.07 | 0.36 | 0.13 |  |
| 3000\|8000 | 0.37 | 0.06 | 0.37 | 0.07 | 0.26 | 0.13 |  |
| 4000\|6000 | 0.55 | 0.07 | 0.54 | 0.06 | 0.51 | 0.10 |  |
| 4000\|8000 | 0.35 | 0.06 | 0.35 | 0.07 | 0.32 | 0.12 |  |
| 6000\|8000 | 0.44 | 0.06 | 0.42 | 0.07 | 0.28 | 0.14 |  |

**Supplemental Table 3. Environmental correlations for frequency pairs.** Environmental variance correlations are shown for: A) all relationships: generations 1, 2, and 3, B) parent and offspring relationships: generations 2 and 3, and C) sibling relationships in generation 3.

**Supplemental Methods**

**Resource Table**

| **Software Package** | **Purpose** | **Reference** |
| --- | --- | --- |
| Sequential Oligogenic  Linkage Analysis  Routines (SOLAR) | Heritability, genetic correlation | (Almasy & Blangero, 1998) |
| PLINK | Data quality control, file preparation | (Chang et al., 2015; Purcell et al., 2007) |
| BCFTOOLS | Genomic file manipulation and quality control | (Danecek et al., 2021) |
| R: statistical software | Data manipulation | (R Core Team, 2024) |

**Computational Software**

**SOLAR**

SOLAR: Sequential Oligogenic Linkage Analysis Routines utilize genotype, phenotype, and familial relationships. This package performs variance component analysis to decompose phenotypic variation into genetic and environmental variance components. Using maximum likelihood estimation, SOLAR estimates heritability, polygenicity, and genetic correlation. This involves optimizing a likelihood function through an iterative approach using variance component data to maximize the likelihood function.
