# Supplementary figures and images for: Genetic resonance: dissecting the heritability and genetic correlations of human hearing acuity
Source: G3 (Bethesda). 2024 Dec 12;15(2):jkae292. doi: 10.1093/g3journal/jkae292 (PMC11797064; doi:10.1093/g3journal/jkae292)

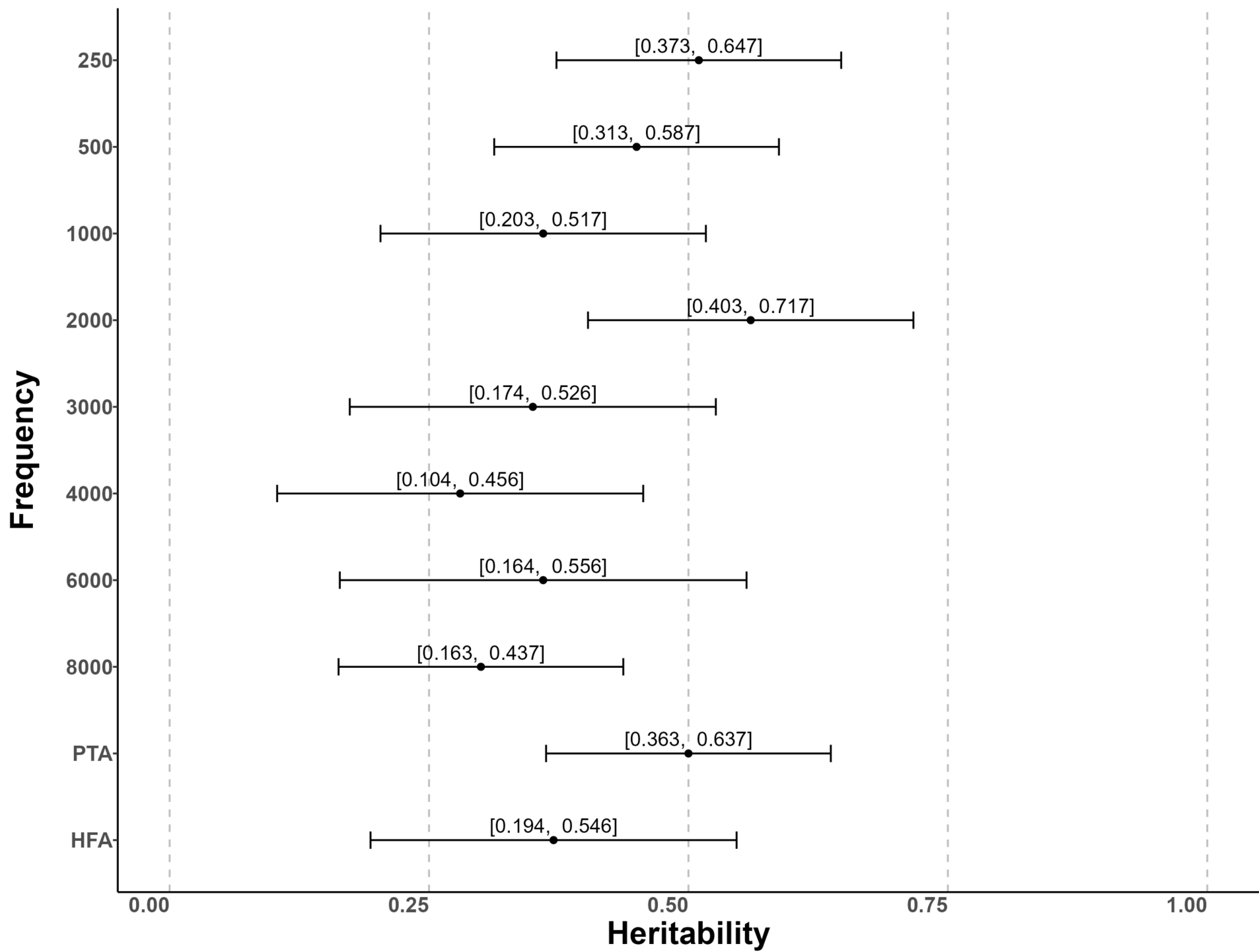

Supplement: jkae292_Supplementary_Data [file jkae292_supplementary_data.zip › Supplemental_Figure_1_G3-2024-405327.pdf]

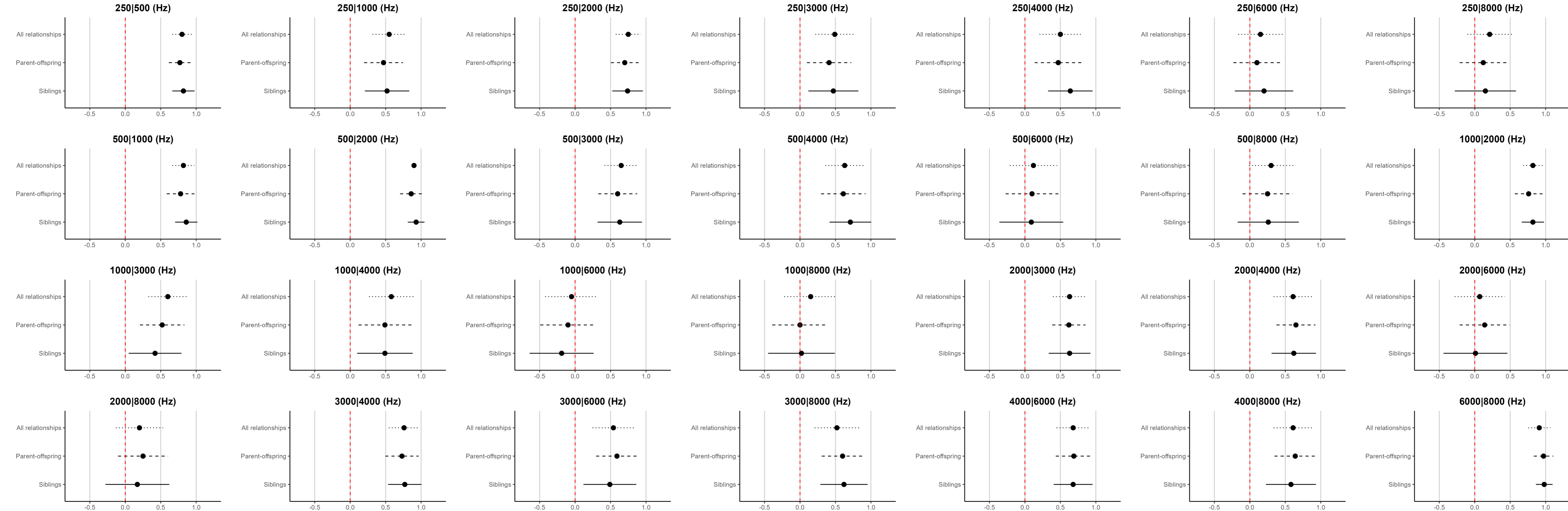

Genetic corretlation

Supplement: jkae292_Supplementary_Data [file jkae292_supplementary_data.zip › Supplemental_Figure_3_G3-2024-405327.pdf]

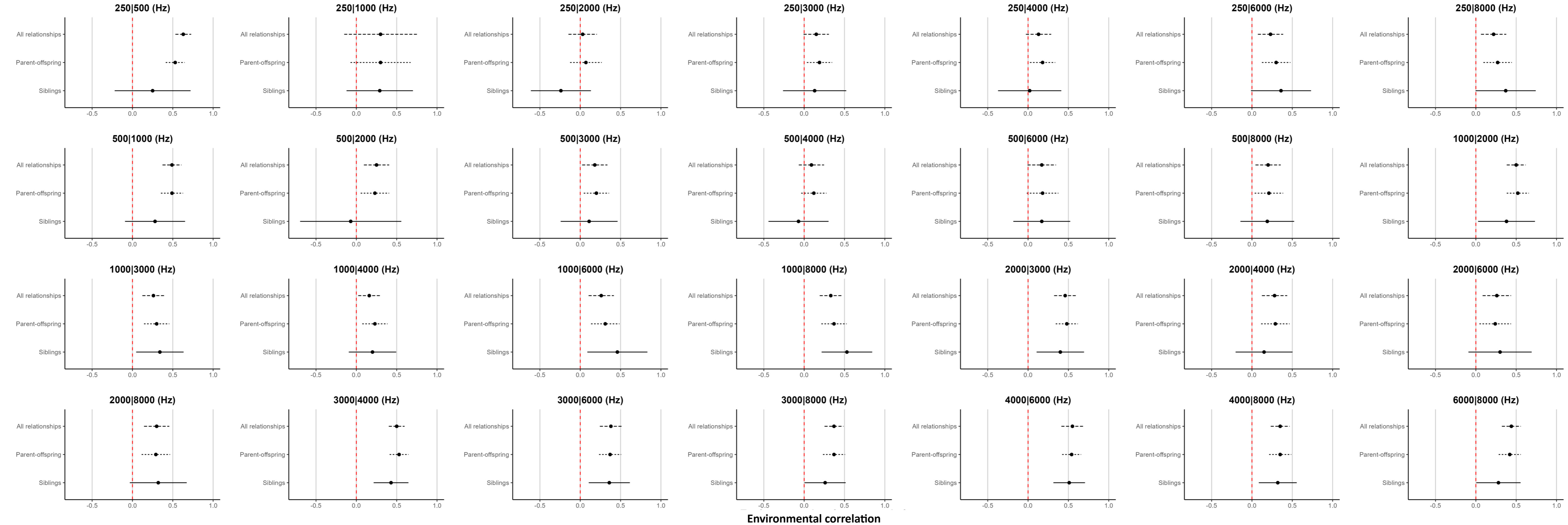

Supplement: jkae292_Supplementary_Data [file jkae292_supplementary_data.zip › Supplemental_Figure_4_G3-2024-405327.pdf]
