# Supplementary material for: Genetic resonance: dissecting the heritability and genetic correlations of human hearing acuity
Source: G3 (Bethesda). 2024 Dec 12;15(2):jkae292. doi: 10.1093/g3journal/jkae292 (PMC11797064; doi:10.1093/g3journal/jkae292)
Supplement: jkae292_Supplementary_Data [file Supplemental_Figure_2_G3-2024-405327.pdf › Supplemental_Figure_2_G3-2024-405327.pdf]

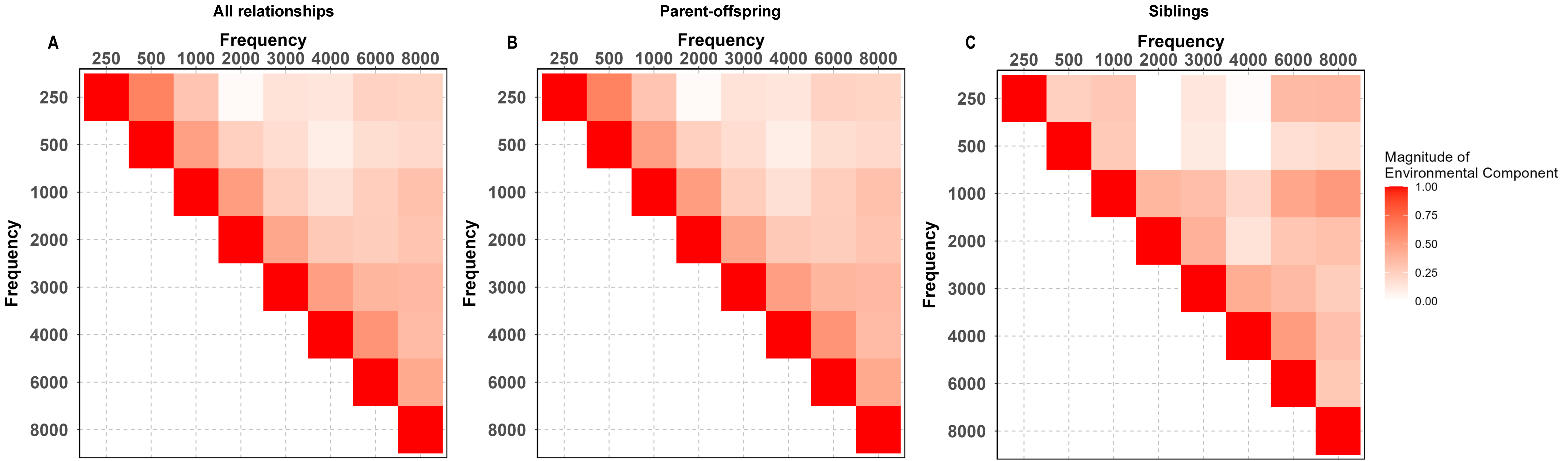

All relationships
Parent-offspring
Siblings
A
B
C
Frequency
250
500
1000
2000
3000
4000
6000
8000
Magnitude of
Environmental Component
1.00
0.75
0.50
0.25
0.00
